# Supplementary material for: Microbiota Composition of Mucosa and Interactions between the Microbes of the Different Gut Segments Could Be a Factor to Modulate the Growth Rate of Broiler Chickens
Source: Animals (Basel). 2022 May 18;12(10):1296. doi: 10.3390/ani12101296 (PMC9137591; doi:10.3390/ani12101296)
Supplement: Supplementary file 1 [file animals-12-01296-s001.zip › animals-1699962-supplementary.pdf]

Microbiota Composition of Mucosa and Interactions between the Microbes of the Different Gut Segments Could Be a Factor to Modulate the Growth Rate of Broiler Chickens

Supplementary Materials

* Corresponding author: Prof. Károly Dublecz

E-mail address: dublecz.karoly@uni-mate.hu

Supplemental Table S1. Composition of diets (starter, grower and finisher) fed to the experimental chicks.

	Starter Diet (day 1-14)	Grower Diet (day 15-32)	Finisher Diet (day 33-37)
**Ingredient (g/kg)**			
Corn	412.50	442.62	468.10
Wheat	150	150	150
Soybean meal	370	243	178
Sunflower oil	25	28	32
Fullfat soybean	0	60	80
Sunflower meal	0	40	60
L-lizin	3.2	2.8	2.3
DL-Metionin	3.76	2.96	2.39
L-Treonin	1.34	0.92	0.51
Limestone	14.5	12	11
MCP	10	8	6.5
Salt	2.7	2.7	2.7
Sodium bicarbonate	1.5	1.5	1.5
Anticoccidian (Sacox 120)	0.5	0.5	0
Avizyme 1505 X	0	0	0
Premix[1]	5	5	5
Calculated nutrient content ()			
AME_n (MJ/kg)[2]	12.50	12.96	13.26
Crude proteins	22.0	20.0	18.6
Lysine	1.44	1.25	1.10
Methionine + cysteine	1.07	0.95	0.86
Tryptophan	0.26	0.23	0.21
Threonine	0.97	0.84	0.74
Calcium	0.92	0.78	0.71
Available P	0.50	0.45	0.42

[1]Vitamin-mineral premix contents per kg: 15 calcium 2.500.000 IU vitamin A, 1.000.000 IU vitamin D3, 16.800 mg vitamin E, 984 mg vitamin K3, 590.4 mg vitamin B1, 1.968 mg vitamin B2, 4.2 mg vitamin B12, 10.000 mg niacin, 2.952 mg pantothenic acid, 393.6 mg Folic acid, 40 mg biotin, 984 mg vitamin B6. 35.070 mg choline chloride, 29.008 mg betaine, 10.016 mg iron, 21.700 mg manganese, 3.200 mg copper, 20.160 mg zinc, 312.33 mg iodine, 63 mg selenium, 1.050 mg antioxidant, 200.000 FTU of Phytase (Axtra PHY).
[2]AMEn values were calculated according to EU regulation 152/2009.

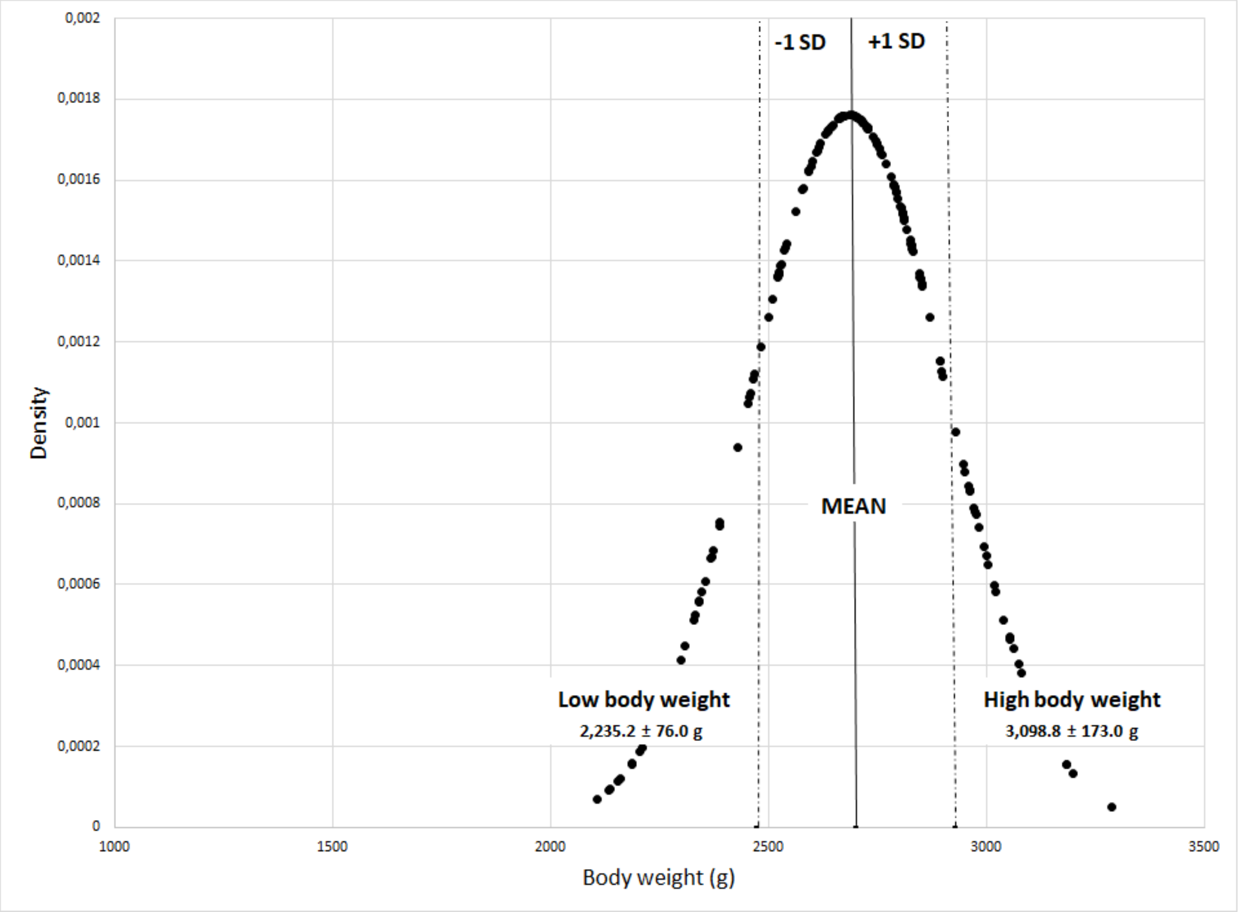

Supplemental Figure S1. Sampling strategy. A total of 160 male broiler chickens were weighed individually and average BW (mean ± SD) were determined. For the selection. Five birds with heaviest body weight (mean BW +1 SD) and five lightest (mean BW -1 SD) were selected randomly and classify into two group with high or low live weight (HBW and LBW).

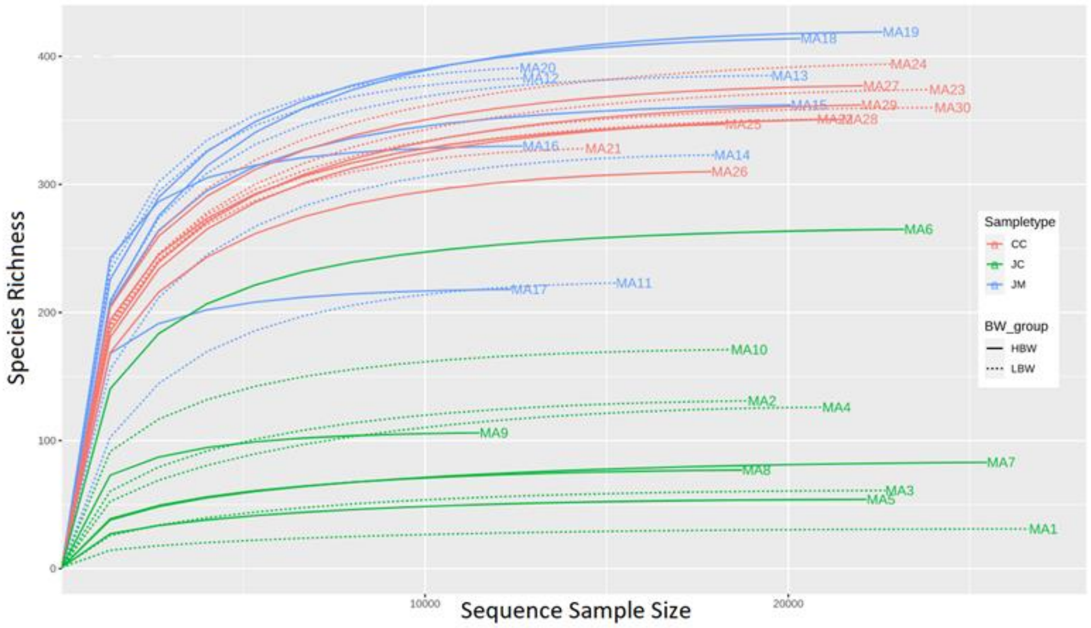

Supplemental Figure S2. The rarefaction curves of OTUs. The x-axis shows the number of valid sequences per sample and the y-axis shows the observed species (operational taxonomic units, OTUs). Each curve in the graph represents a different sample and the sample types shows in a different color, BW group indicates a solid (HBW) and dashed (LBW) line.

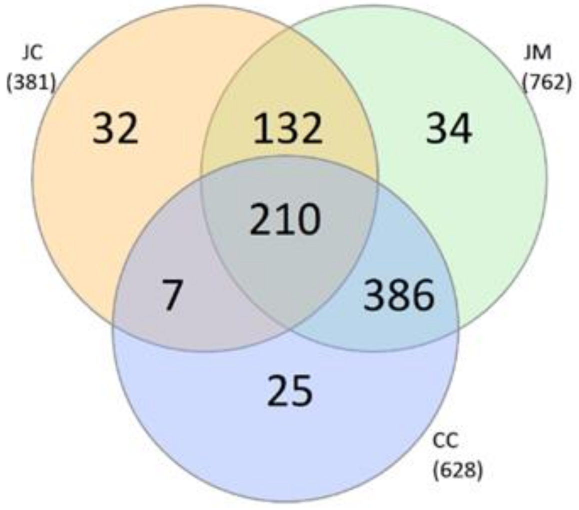

Supplemental Figure S3. Venn diagram show the number of OTUs that were shared or not shared in the jejunum mucosa (JM), jejunum content (JC), and caecum content (CC), or overlapped in the 3 examined groups.

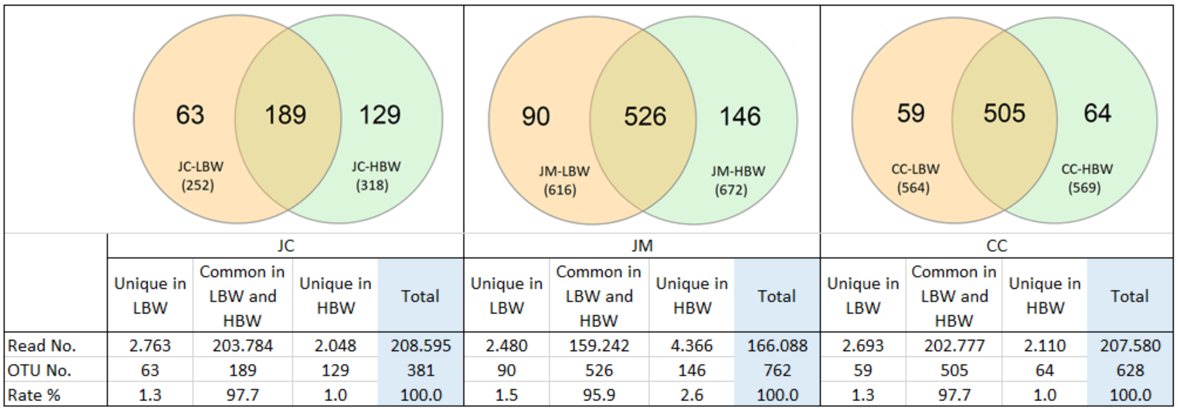

	JC				JM				CC			
	Unique in LBW	Common in LBW and HBW	Unique in HBW	Total	Unique in LBW	Common in LBW and HBW	Unique in HBW	Total	Unique in LBW	Common in LBW and HBW	Unique in HBW	Total
Read No.	2.763	203.784	2.048	208.595	2.480	159.242	4.366	166.088	2.693	202.777	2.110	207.580
OTU No.	63	189	129	381	90	526	146	762	59	505	64	628
Rate %	1.3	97.7	1.0	100.0	1.5	95.9	2.6	100.0	1.3	97.7	1.0	100.0

Supplemental Figure S4. Venn diagrams shows the number of OTUs in different intestinal sampling places according to the body weight (low and high BW) of chickens indicating in table their read numbers and rates (%).

Supplemental Table S2. Relative abundance of bacterial genera in the different sampling places of broiler chickens as affected by BW (%).

Genus	Body Weight	Sampling Place			Mean (BW)	FDR p-Values		
		Jejunal Chymus	Jejunal Mucosa	Caecum Chymus		SP	BW	SP x BW
**Actinobacteria**								
Corynebacterium-1	LBW	2.06	0.97	0.00	**1.01**			0.747
	HBW	3.51	0.24	0.01	**1.25**		0.877	
	**Mean (SP)**	**2.78**	**0.60**	**0.00**		0.323		
**Bacteroidetes (Class Bacteroidia)**								
Alistipes	LBW	0.00	0.64	0.82^{B}	**0.48^{B}**			0.451
	HBW	0.03	1.71	2.48^{A}	**1.41^{A}**		*0.081^{T}*	
	**Mean (SP)**	**0.01^{b}**	**1.17^{a}**	**1.65^{a}**		0.017		
Bacteroides	LBW	0.11	19.06^{B}	42.39	**20.52^{B}**			0.280
	HBW	0.76	34.28^{A}	46.27	**27.10^{A}**		*0.098^{T}*	
	**Mean (SP)**	**0.43^{c}**	**26.67^{b}**	**44.33^{a}**		0.000		
Parabacteroides	LBW	0.00	0.58	1.99	**0.86**			0.509
	HBW	0.04	1.97	2.69	**1.56**		0.179	
	**Mean (SP)**	**0.02^{c}**	**1.27^{b}**	**2.34^{a}**		0.000		
**Firmicutes (Class Bacilli)**								
Bacillus	LBW	0.01	0.55	0.13	**0.23**			0.948
	HBW	0.03	0.65	0.09	**0.25**		0.907	
	**Mean (SP)**	**0.02^{b}**	**0.60^{a}**	**0.11^{b}**		0.024		
Enterococcus	LBW	0.02^{B}	0.03	0.00	**0.02^{B}**			0.001
	HBW	0.22^{A}	0.02	0.00	**0.08^{A}**		0.012	
	**Mean (SP)**	**0.123^{a}**	**0.025^{b}**	**0.002^{b}**		0.000		
Lactobacillus	LBW	65.94	21.87	2.90	**30.24**			0.721
	HBW	64.24	7.23	7.23	**25.07**		0.646	
	**Mean (SP)**	**65.09^{a}**	**14.55^{b}**	**3.32^{b}**		0.000		
Streptococcus	LBW	7.56	1.01	4.32	**4.30**			0.690
	HBW	6.77	1.05	0.96	**2.93**		0.585	
	**Mean (SP)**	**7.17^{a}**	**1.03^{b}**	**2.64^{b}**		0.022		
Nosocomiicoccus	LBW	0.15	0.06	0.00	**0.07**			0.906
	HBW	0.10	0.04	0.00	**0.05**		0.680	
	**Mean (SP)**	**0.12**	**0.05**	**0.00**		0.208		
Lactococcus	LBW	0.10	0.00	0.00	**0.04**			0.595
	HBW	1.36	0.00	0.00	**0.45**		0.585	
	**Mean (SP)**	**0.73**	**0.00**	**0.00**		0.513		
**Firmicutes (Class Clostridia; Families Family XIII and Christensenellaceae)**								
Family XIII UCG-001	LBW	0.00	0.06	0.08	**0.05**			0.624
	HBW	0.00	0.13	0.15	**0.09**		0.323	
	**Mean (SP)**	**0.00^{b}**	**0.10^{a}**	**0.12^{a}**		0.013		
Christensenellaceae R-7	LBW	0.03	0.64	1.39	**0.68**			0.476
	HBW	0.01	0.93	1.12	**0.69**		0.989	
	**Mean (SP)**	**0.02^{c}**	**0.78^{b}**	**1.25^{a}**		0.000		
**Firmicutes (Class Clostridia; Family Lachnospiraceae)**								
Anaerostipes	LBW	0.01	0.12	0.13	**0.09**			0.500
	HBW	0.00	0.17	0.06	**0.08**		0.821	
	**Mean (SP)**	**0.00^{b}**	**0.14^{a}**	**0.10^{a}**		0.007		
Blautia	LBW	0.03	0.23	0.59	**0.28**			0.417
	HBW	0.03	0.30	0.35	**0.23**		0.623	
	**Mean (SP)**	**0.03^{c}**	**0.26^{b}**	**0.47^{a}**		0.000		
CHKCI001	LBW	0.01	0.04	0.26	**0.11**			0.989
	HBW	0.02	0.03	0.28	**0.11**		0.955	
	**Mean (SP)**	**0.01^{b}**	**0.04^{b}**	**0.27^{a}**		0.024		
Eubacterium hallii group	LBW	0.02	0.17	0.56	**0.25**			0.985
	HBW	0.02	0.09	0.49	**0.20**		0.796	
	**Mean (SP)**	**0.02**	**0.13**	**0.53**		0.067		
Eubacterium ventriosum	LBW	0.00	0.02	0.10	**0.04**			0.919
	HBW	0.00	0.03	0.11	**0.05**		0.727	
	**Mean (SP)**	**0.00^{b}**	**0.02^{b}**	**0.11^{a}**		0.000		
GCA-900066575	LBW	0.00	0.26	0.14	**0.13**			0.680
	HBW	0.00	0.21	0.05	**0.09**		0.456	
	**Mean (SP)**	**0.00^{c}**	**0.24^{a}**	**0.09^{b}**		0.000		
Lachnoclostridium	LBW	0.02	0.65	1.12	**0.59**			
	HBW	0.05	0.62	1.10	**0.59**		0.989	

	**Mean (SP)**	**0.03 ^{c}**	**0.64 ^{b}**	**1.11 ^{a}**		0.000		0.989
Ruminococcus torques group	LBW	0.05	1.82	1.81	**1.23**			0.624
	HBW	0.08	2.39	1.12	**1.20**		0.970	
	**Mean (SP)**	**0.06 ^{b}**	**2.11 ^{a}**	**1.46 ^{a}**		0.008		
Sellimonas	LBW	0.01	0.32	0.49	**0.27**			0.991
	HBW	0.02	0.31	0.49	**0.27**		0.995	
	**Mean (SP)**	**0.02 ^{c}**	**0.31 ^{b}**	**0.49 ^{a}**		0.000		
Tyzzerella	LBW	0.00	0.19	0.29	**0.16**			0.610
	HBW	0.00	0.40	0.27	**0.22**		0.624	
	**Mean (SP)**	**0.00 ^{b}**	**0.29 ^{a}**	**0.28 ^{a}**		0.025		
**Firmicutes (Class Clostridia; Family Ruminococcaceae)**								
Anaerofilum	LBW	0.00	0.17	0.09	**0.09**			0.885
	HBW	0.02	0.18	0.07	**0.09**		0.962	
	**Mean (SP)**	**0.01 ^{b}**	**0.17 ^{a}**	**0.08 ^{b}**		0.003		
Butyricicoccus	LBW	0.02	0.49	0.85	**0.45**			0.327
	HBW	0.05	0.45	0.52	**0.34**		0.346	
	**Mean (SP)**	**0.03 ^{c}**	**0.47 ^{b}**	**0.69 ^{a}**		0.000		
DTU089	LBW	0.01	0.24	0.16	**0.14**			0.699
	HBW	0.00	0.15	0.10	**0.08**		0.372	
	**Mean (SP)**	**0.00 ^{b}**	**0.20 ^{a}**	**0.13 ^{a}**		0.002		
Eubacterium-coprostanoligenes-group	LBW	0.00	1.07	1.03	**0.70**			0.810
	HBW	0.02	1.27	0.99	**0.76**		0.740	
	**Mean (SP)**	**0.01 ^{b}**	**1.17 ^{a}**	**1.01 ^{a}**		0.000		
Faecalibacterium	LBW	0.01	3.15	3.08	**2.08**			0.683
	HBW	0.00	2.45	1.33	**1.26**		0.503	
	**Mean (SP)**	**0.01 ^{b}**	**2.80 ^{a}**	**2.20 ^{a}**		0.031		
Fournierella	LBW	0.00	0.29	0.24	**0.18**			0.417
	HBW	0.02	0.61	0.28	**0.30**		0.323	
	**Mean (SP)**	**0.01 ^{b}**	**0.45 ^{a}**	**0.26 ^{a}**		0.001		
Negativibacillus	LBW	0.00	0.17	1.13 ^{A}	**0.44**			0.027
	HBW	0.00	0.20	0.59 ^{B}	**0.26**		0.127	
	**Mean (SP)**	**0.00 ^{b}**	**0.19 ^{b}**	**0.86 ^{a}**		0.000		
Oscillibacter	LBW	0.00	0.66	0.70	**0.46**			0.690
	HBW	0.00	0.89	0.69	**0.53**		0.660	
	**Mean (SP)**	**0.00 ^{b}**	**0.77 ^{a}**	**0.70 ^{a}**		0.000		
Ruminiclostridium 5	LBW	0.00	0.28	0.44	**0.24**			0.260
	HBW	0.00	0.38	0.23	**0.21**		0.665	
	**Mean (SP)**	**0.00 ^{b}**	**0.33 ^{a}**	**0.34 ^{a}**		0.000		
Ruminococcaceae NK4A214	LBW	0.01	0.35	0.82	**0.40**			0.342
	HBW	0.01	0.44	1.79	**0.75**		0.329	
	**Mean (SP)**	**0.01 ^{b}**	**0.39 ^{b}**	**1.31 ^{a}**		0.001		
Ruminococcaceae UCG-005	LBW	0.01	1.03	0.70	**0.58**			0.727
	HBW	0.01	1.51	0.70	**0.74**		0.665	
	**Mean (SP)**	**0.01 ^{b}**	**1.27 ^{a}**	**0.70 ^{ab}**		0.009		
Ruminococcaceae UCG-010	LBW	0.00	0.09 ^{B}	0.19	**0.09 ^{B}**			0.472
	HBW	0.00	0.24 ^{A}	0.29	**0.18 ^{A}**		*0.083 ^{T}*	
	**Mean (SP)**	**0.00 ^{b}**	**0.17 ^{a}**	**0.24 ^{a}**		0.000		
Ruminococcaceae UCG-013	LBW	0.01	0.09	0.39 ^{A}	**0.17 ^{A}**			0.026
	HBW	0.01	0.06	0.18 ^{B}	**0.08 ^{B}**		0.020	
	**Mean (SP)**	**0.010 ^{b}**	**0.078 ^{b}**	**0.285 ^{a}**		0.000		
Ruminococcaceae UCG-014	LBW	0.02	2.47	3.46	**1.98**			0.690
	HBW	0.15	3.70	4.79	**2.88**		0.341	
	**Mean (SP)**	**0.09 ^{b}**	**3.09 ^{a}**	**4.12 ^{a}**		0.000		
Subdoligranulum	LBW	0.04	0.29	0.90	**0.41**			0.803
	HBW	0.14	0.64	1.11	**0.63**		0.371	
	**Mean (SP)**	**0.09 ^{b}**	**0.46 ^{b}**	**1.01 ^{a}**		0.000		
**Firmicutes (Class Negativicutes and Erysipelotrichia)**								
Phascolarctobacterium	LBW	0.03	1.38	3.98	**1.80**			0.607
	HBW	0.03	1.01	2.06	**1.03**		0.475	
	**Mean (SP)**	**0.03 ^{b}**	**1.19 ^{b}**	**3.02 ^{a}**		0.008		
Erysipelatoclostridium	LBW	0.00	0.22	0.68	**0.30**			0.381
	HBW	0.01	0.01	0.38	**0.16**		0.112	
	**Mean (SP)**	**0.01 ^{b}**	**0.15 ^{b}**	**0.53 ^{a}**		0.000		
**Proteobacteria**								
Burkholderia-Caballeronia-Paraburkholderia	LBW	0.00	0.11	0.00	**0.26**			0.471
	HBW	0.00	0.10	0.00	**0.19**		0.630	
	**Mean (SP)**	**0.00 ^{b}**	**0.11 ^{a}**	**0.00 ^{b}**		0.036		
Enterobacter	LBW	0.06	0.45	0.00	**0.25**			0.985

	HBW	0.02	0.02	0.00	**0.20**		0.796	
	**Mean (SP)**	**0.02**	**0.13**	**0.53**		0.067		
Escherichia-Shigella	LBW	0.98	0.65	1.79	**1.14**			0.157
	HBW	8.18	0.37	0.64	**3.06**		0.450	
	**Mean (SP)**	**4.58**	**0.51**	**1.22**		0.190		
Paracoccus	LBW	0.01	0.09	0.00	**0.03**			0.683
	HBW	0.12	0.09	0.00	**0.07**		0.646	
	**Mean (SP)**	**0.064**	**0.089**	**0.00**		0.577		
Bilophila	LBW	0.02	0.23	0.53	**0.04**			0.989
	HBW	0.00	0.33	0.23	**0.03**		0.929	
	**Mean (SP)**	**0.01** [b]	**0.28** [a]	**0.38** [a]		0.017		

Bacterial genera differences between groups were assessed using two-way ANOVA test, with Benjamini-Hochberg false discovery rate (FDR) correction. FDR-corrected p-values below 0.05 were considered significant and results between 0.05 and 0.1 ($0.05 < p < 0.10$) were considered a trend (T). Body weight (BW) effect at each sampling places (SP) were also compared with one-way ANOVA test and the significance of Tukey's HSD multiple group comparison's Post hoc tests was indicated at $p < 0.05$. a,b,c: values within the mean (SP) rows with different lowercase letters were significantly different ($p < 0.05$). A, B: values within the mean columns with different capital letters superscripts were significantly different ($p < 0.05$).

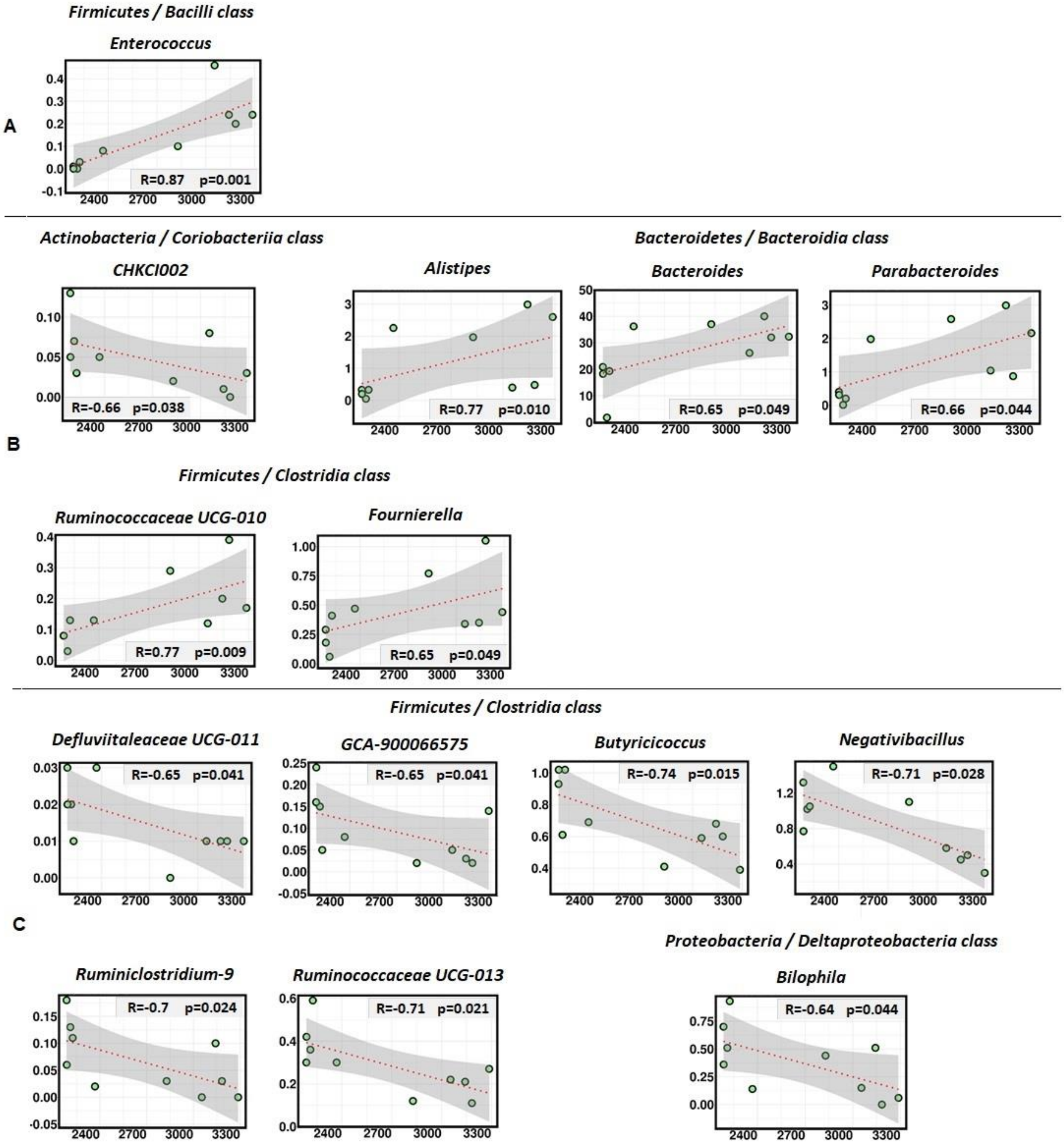

Supplemental Figure S5. Correlation between body weight (g) and genus abundance in the different intestinal samples. (A) Correlation in jejunum chymus; (B) in jejunum mucosa and (C) in caecum chymus.
